# Role of IL-9 and STATs in hematological malignancies (Review)

**DOI:** 10.3892/ol.2013.1761

**Published:** 2013-12-16

**Authors:** NA CHEN, XIN WANG

**Affiliations:** 1 Department of Hematology, Provincial Hospital Affiliated to Shandong University, Jinan, Shandong 250021, P.R. China; 2 Department of Diagnostics, Shandong University School of Medicine, Jinan, Shandong 250012, P.R. China

**Keywords:** hematological malignancies, IL-9, STAT3, STAT6

## Abstract

Although interleukin-9 (IL-9) exhibits pleiotropic functions in the immune system, it remains a well-known cytokine in hematological malignancies. Previous cell culture and animal model studies have revealed that the Janus kinase-signal transducer and activator of transcription signaling pathway, which may be activated by a number of cytokines including IL-9, is critical in hematological malignancies. The current review summarizes the characterization of the biological activities of IL-9, highlights the clearly defined roles of the cytokine, and outlines questions with regard to the functions of IL-9 that require further exploration and their downstream signaling proteins, signal transducers and activators of transcription.

## 1. Introduction

Interleukin (IL)-9 is a member of the common γ-chain family of cytokines, using the γ-chain receptor in combination with the cytokine-specific receptor, IL-9 receptor (IL-9R) α (1). There has been renewed interest in IL-9 since the identification of a subset of T cells which produce this cytokine. However, previous conflicting studies have been identified, concerning which T cells produce this cytokine. The studies demonstrated, but are not limited to, the following T cells: T helper (Th) 2, 9 and 17 cells and regulatory T (Treg) cells. Besides the role of IL-9 during immune responses, its growth factor and antiapoptotic activities on multiple transformed cells suggest a potential role in hematological malignancies. Notably, IL-9 overexpression induces thymic lymphomas in mice, and IL-9 production has an effect on Hodgkin’s disease and human T-lymphotropic virus type I (HTLV-I)-transformed T cells in humans. IL-9 activities also involve IL-2, -4, -7, -15 and -21 signaling, which is mediated by a specific receptor chain that forms a heterodimeric receptor with the common γ chain (2). The IL-9R and common γ chains associate with Janus kinase (JAK) 1 and JAK3 and trigger the signal transducer and activator of transcription (STAT)-1, -3 and -5, insulin receptor signaling (IRS) and RAS-mitogen-activated protein kinase (MAPK) pathways. In addition, IL-9 is not expressed by Th 2 and 9 cells in the absence of STAT6 expression. Dysregulated IL-9 response also leads to autonomous cell growth and the malignant transformation of lymphoid cells associated with constitutive activation of the JAK/STAT pathway *in vitro*. The current review summarizes the characterization of the biological activities of IL-9, highlights the clearly defined roles of the cytokine, and outlines questions with regard to the functions of IL-9 that require further exploration and their downstream signaling proteins, STATs.

## 2. IL-9 production and function

### IL-9-secreting cells

Initially, IL-9 was described as a T cell-derived cytokine with pleiotropic activities on various cell types. IL-9 is mainly expressed by activated CD4^+^ T cells, including Th2, Th9, Th17 and Treg cells (3). As compared with plate-bound anti-CD3 mAb/soluble anti-CD28 mAb plus transforming growth factor (TGF)-β stimulation, IL-4 and TGF-β stimulate memory CD4(+), CD25(−) and CD45RO(+) T cell expression, inducing higher levels of IL-9 expression, but reducing forkhead box (Fox) p3 protein expression. IL-4 and TGF-β inhibit the expression of Foxp3 that induces Treg generation and promotes an IL-9-secreting phenotype, which is dependent on STAT6. Similarly, Gata3 is required for the generation of IL-9-secreting cells, suggesting that the IL-9-secreting population has shared factors in their development. Human T cells also acquire IL-9-secreting potential when cultured with TGF-β and IL-4. IL-9 production was first associated with the Th2 phenotype (4). However, it was unclear whether specialized cells were responsible for IL-9 production and secretion, as was being established at the time for other cytokines, including IL-4 and interferon (IFN) γ, in Th2 and Th1 cells, respectively.

Studies have shown that two transcription factors are required for IL-9-secreting cells and have been reported to bind directly to the *Il9* gene. PU.1 is an ETS-family transcription factor. PU.1-deficient T cells exhibit diminished IL-9 production and ectopic expression of PU.1 increases IL-9 production from Th2 or Th9 cultures (5–7). PU.1 binds directly to the *Il9* gene and histone modifications associated with the Th9 phenotype are dependent upon PU.1. PU.1-specific small interfering RNA results in impaired IL-9 production by human T cells; therefore, PU.1 is also important for IL-9 production in human T cells. Notably, PU.1 is expressed in greater amounts in cells cultured under Th9 conditions compared with Th2 cells, which suggests that PU.1 is a critical factor in diverting Th2 cells into an IL-9-secreting lineage (7). In a previous follow-up study, in the absence of PU.1, a decreased association between Gcn5 and p300/CREB-binding protein (CBP) associated factor and inhibition of the expression of Gcn5 was found to result in reduced IL-9 production, which suggested that Gcn5 may be important in PU.1-dependent IL-9 production. Similar to PU.1, IFN-regulatory factor 4 (IRF4) was also shown to be required for Th9 generation and, possibly in concert with PU.1, since IRF4 was originally identified as a PU.1-interacting protein, IRF4 binds to the *Il9* gene directly. In addition, IRF4 expression has been associated with human and mouse Th9 differentiation and is induced by cytokines that promote IL-9 production, including IL-4, IL-2 and TGF-β (8). IRF4 is important in the cytokine-secreting potential of several Th subsets as IRF4 is also required for Th2 and Th17 development (9–11).

**Figure 1:**
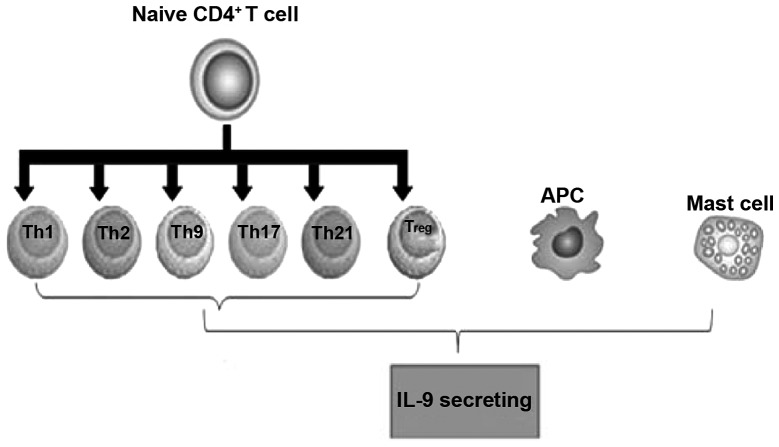
IL-9-secreting cells. IL-9, interleukin-9; Th, T-helper; Treg, regulatory T; APC, antigen presenting cell.

Mast cells (MCs) also produce IL-9 in response to lipopolysaccharides and IL-1, which are associated with the presence of nuclear factor κB (NF-κB) binding sites in the *Il9* promoter that mediate gene activation (12–14). Gata1 in MCs promotes IL-9 production and *Il9* promoter activation is dependent upon p38 MAPK (15). MCs and T cells, as well as the scenarios in which each cell may contribute to IL-9 production *in vivo*, have not previously been determined. In addition, IL-9 induces MC production of TGF-β, which exhibits proinflammatory downstream effects (Fig. 1).

### IL-9R signaling and expression

The functions of IL-9 are mediated by the IL-9R, which is a member of the hematopoietin receptor superfamily (16). The IL-9R is shared with the IL-2, -4, -7, -15 and -21 receptors, including the ligand-specific α chain and the common γ chain. The mouse receptor contains 468 amino acids, but the human IL-9R gene contains 11 exons and encodes a 522-amino acid protein. On the basis of the presence of the WSXWS motif in the extracellular domain and Box1 and Box2 motifs in the intracellular domain, the IL-9Rα is a member of the hematopoietin superfamily. The Box1 intracellular domain is critical for IL-9-induced cell growth, activation of STAT3 and induced gene expression, which is between amino acids 338 and 422, including a YLPQ motif. Although IL-9Rα expression is not induced by Tax, expression of IL-9 is activated by Tax via an NF-κB motif in its proximal promoter.

The IL-9R and common γ chains associated with JAK1 and JAK3, trigger STATs-1, -3 and -5. In addition, they activate the IRS and RAS-MAPK pathways, although, the physiological requirement for these pathways in primary cells has not previously been well documented (17). A single tyrosine residue (Tyr407) in the IL-9Rα is phosphorylated following ligand binding to the receptor and activation of associated JAK1. The mutation of this residue demonstrates that it is required for IL-9-dependent responses.

As predicted from its initial identification as a T-cell growth factor, IL-9R is expressed in T-cell lines and effector T cells, but not in naïve T cells (18,19). Among the Th-cell subsets, IL-9R exhibits its highest expression in Th2 and Th17 cells (13). IL-9R is found on MCs and polymorphonuclear leukocytes (20,21). Additionally, IL-9R is expressed in non-hematopoietic cells. Since γc is unlikely to be expressed in these cells, the exact composition of the receptor in non-hematopoietic cells has not been clearly defined.

### Effect of IL-9 on B cells

Traditionally, IL-9 is described as a T-cell-derived cytokine with pleiotropic activities on various cell types. More recently, IL-9 expression has been linked to B cells (22,23). IL-9 not only exerts effects on B-cell development but also on function. Transgenic expression of IL-9 recovers the B1 cell numbers, but not natural IgM production, and results in an increase of peritoneal CD11b^+^ B1 cells, in *xid* mice (24,25). IL-9 enhances IL-4-mediated IgE and IgG production from human B cells, but has no effect on IgM production (26,27). IL-9 exerts similar effects on germinal center B cells and IL-9Rα expression is greater on such B cells than on other types of B cells (28).

### IL-9-dependent regulation of hematological malignancies

IL-9 is a T-cell-derived lymphokine that induces the proliferation of various lymphoid and hemopoietic cells (29). The HTLV-I protein, Tax, is important in the early stages of adult T-cell leukemia/lymphoma (ATL) based on altered gene expression, including that of cytokines and their receptors. Supporting a role for IL-9/IL-9Rα in ATL, a previous study showed that a neutralizing monoclonal antibody inhibited the *ex vivo* spontaneous proliferation of primary ATL cells obtained from several patients, directed toward IL-9Rα. Freshly isolated peripheral blood mononuclear cells from these patients revealed high expression levels of IL-9Rα on their CD14-expressing monocytes by fluorescence-activated cell sorting analysis. Furthermore, purified T cells or monocytes did not independently proliferate from these patients *ex vivo*, whereas mixtures of these cell types manifested significant proliferation in a contact-dependent manner. Overall, these results suggested that primary ATL cells support the action of IL-9Rα/CD14-expressing monocytes via IL-9, which subsequently supports the *ex vivo* spontaneous proliferation of malignant T cells. In conclusion, these results supported the theory that IL-9 and its receptor are involved in ATL through a paracrine mechanism (17,30).

Previous studies analyzing the culture supernatants of peripheral blood mononucleated cells (PBMCs) from the early phases of ATL patients with spontaneous proliferation using the cytokine-dependent indicator cell line, NK-92, have revealed that the majority of six-day culture supernatants of PBMCs from ATL patients contain high amounts of IL-9 (31). The presence of IL-9 in the culture supernatants was also confirmed by ELISA analysis. Furthermore, in certain ATL patients within this group, the spontaneous proliferation was blocked by a monoclonal antibody against IL-9Rα. This suggested that the IL-9/IL-9R system may be involved in the expansion of the HTLV-I-infected CD4^+^ T cells of specific patients in the early stages of ATL (32). IL-9 is a Th2 cytokine and IL-9 is produced in >80% of smoldering/chronic ATL *ex vivo* PBMC cultures, which has previously shown that HTLV-1 Tax transactivates IL-9 expression in HTLV-1-infected T-cell lines (30). Prevention of histone acetylation by the histone acetyltransferase inhibitor, curcumin, diminished PU.1 expression following IL-9-inducing stimulation. Autocrine/paracrine cytokine stimulation of leukemic cell proliferation has been identified in patients with smoldering/chronic ATL that may be targeted for treatment. The etiologic agent of ATL is HTLV-I.

Anaplastic lymphoma kinase (ALK) has been implicated in the growth of neoplastic cells in malignant lymphomas. Although IL-9 has been implicated in the growth of normal MCs, little is known concerning pro-oncogenic molecules and conditions triggering differentiation and growth of MC to lead to the histopathological image of overt mastocytosis. Certain previous studies have described that transplantation of nucleophosmin (*NPM)-ALK*-transplanted mouse bone marrow progenitors into lethally irradiated IL-9 transgenic mice not only results in lymphoma formation, but also in the development of a neoplastic disease exhibiting histopathological features of systemic mastocytosis. These include multifocal dense MC infiltrates, occasionally with devastating growth in visceral organs. Transplantation of *NPM-ALK*-transduced progenitors into normal mice or maintenance of IL-9 transgenic mice without NPM-ALK both resulted in MC hyperplasia, but not in mastocytosis. Neoplastic MCs in mice are not only exhibited in IL-9, but also the IL-9R, as was found in human neoplastic MCs. Overall, the data show that neoplastic MCs express IL-9Rs. In addition, IL-9 and NPM-ALK upregulate MC production *in vivo* and the two ‘hits’ act in concert to induce a mastocytosis-like disease in mice (33). These results may have pathogenetic and clinical implications, and are consistent with the observation that neoplastic MCs in advanced systemic mastocytosis markedly express NPM and multiple ‘lymphoid’ antigens, including CD25 and CD30.

## 3. Characteristics and expression of STATs

### STAT3 target genes

STAT3 is a cytoplasmic transcription factor and a member of the STAT family. Aberrant STAT3 activation is considered a molecular abnormality that supports the tumor phenotype and is detected with high frequency in hematological malignancies. STAT3 is involved in embryonic stem (ES) cell self-renewal (stemness) of certain mammalian cell types and species. Generally, STAT3-mediated transcription directs cells into cell survival and cell cycle progression. STAT3 is involved in cellular transformation and tumorigenesis (34). However, the molecular mechanisms leading to the aberrant STAT3 activation and STAT3-mediated transformation and tumorigenesis remain unclearly defined.

STAT3 proteins are also activated via cytoplasmic kinases of the Src kinase family and via the tyrosine kinase activity of various growth factor receptors. Cytokines activate STAT3 proteins differentially, utilizing the gp130 receptor component, including ciliary neurotrophic factor, oncostatin M, leukemia-inhibitory factor (LIF) and IL-6. STAT3 proteins are also activated by growth hormones, including thrombopoietin, granulocyte colony-stimulating factor, granulocyte-macrophage colony-stimulating factor, basic fibroblast growth factor and a number of ILs (35). The results of previous proteomic studies have uncovered an interdependence of STAT3 signaling and members of the Rho family of small GTPases, including Rac1, Cdc42 and RhoA. Specifically, Rac1, acting in complex with the male germ cell, RacGAP, promotes tyrosine phosphorylation of STAT3 by the IL6-receptor family/JAK complex, as well as its translocation to the nucleus. Evidence has further demonstrated that the mutational activation of Rac1 and Cdc42 results in STAT3 activation, which occurs, in part, through the upregulation of the IL6 family of cytokines that, in turn, stimulate STAT3 through JAKs. Notably, previous studies have also shown that the engagement of cadherins and cell-to-cell adhesion molecules, specifically, induce a marked increase in Rac1 and Cdc42 protein levels and activity, which, in turn, results in STAT3 activation (36).

STAT3 is tightly integrated into the gene regulatory mechanism of pluripotency. Previously, Kidder *et al* (37) applied chromatin immunoprecipitation (ChIP) sequencing technology to use promoter arrays of mouse ES cells (mESCs) that cover 28,000 promoter regions in the genome. The authors found 948 putative target genes for STAT3, with 29 of these genes co-occupied by *Oct4* and *Nanog*. In addition, Chen *et al* (38) used the ChIP-chip approach to map the binding sites for STAT3 in mESCs. The authors identified 2,546 genomic sites where STAT3 was bound and approximately one-third (718) of these loci were bound by Oct4, Sox2 and Nanog.

The target gene list contains transcriptionally active and inactive genes. A number of pluripotency genes, such as *Oct4* and *Nanog*, exist among the STAT3-binding sites (39). The inactive genes include developmentally regulated tissue-specific genes, including *Gata3* (ectoderm-specific), *Foxa2*, *Gata4* (the two endoderms), *T* (brachyury), LIM homeobox protein 1 (mesoderm) and *Eomes* (trophectoderm). Although the present review did not identify whether STAT3 suppresses these genes, it is possible that STAT3 mediates the suppression of differentiation genes and that this mechanism may be one method in which LIF prevents the differentiation of mESCs into endoderm and mesoderm lineages (40). In a previous study, although the authors did not report whether any of these 113 suppressed genes included the aforementioned developmentally regulated genes, it was found that STAT3 was bound to 113 genes that were also bound by subunits of the polycomb repressive complex 2, such as Suz12 and Eed (37). In general, STAT proteins function as transcriptional activators, but STAT1 is known to suppress the transcription of matrix metalloproteinases and cell-cycle genes (c-Myc, cyclin D and cyclin A) (41). Therefore, STAT3 may also function as a suppressor. Along an associated line of inquiry, Bourillot *et al* (42) knocked down 22 STAT3 target genes and found that 16 induced activation of endodermal genes and one activated mesodermal genes. These observations are consistent with the theory that STAT3 contributes to the prevention of mESC differentiation by suppressing lineage-specific genes.

### STAT3-binding proteins and gene activation

An additional method to understanding how STAT3 regulates gene expression is to identify proteins that interact with STAT3. The interacting proteins include the transcription factors, NF-κB (43) and c-Jun (44), the coactivators, nuclear receptor coactivator 1/SRC1a (45) and Ctr9 (46), and the chromatin remodeling adenylpyrophosphatase (ATPase) brahma-related gene 1 (Brg1) (47,48), p300/CBP. These interactions have been previously studied outside the field of stem cell biology, but similar interactions are likely to occur in mESCs since specific interacting partners are colocalized with STAT3 on the mESC genome. The transactivation domain of STAT3 at the C-terminus interacts with a number of chromatin proteins, including p300/CBP and is colocalized with STAT3 on a number of pluripotency genes (38).

Of particular interest is Brg1, a catalytic subunit of the switch/sucrose non-fermentable ATPase complex that is associated with pluripotency at several levels. Brg1 is involved in chromatin relaxation (99); however, the role of Brg1 in mESCs is not limited to chromatin relaxation. Previously, it was demonstrated that the ESC-specific chromatin remodeling esBAF complex contains Brg1 (50,51). esBAF is colocalized with STAT3 throughout the genome, including in pluripotency genes. Although esBAF binds to a number of pluripotency genes in ESCs, it maintains pluripotency primarily by suppressing differentiation-specific genes (49). Additionally, Brg1 facilitates the binding of *Oct4* to its target genes and increases the efficiency of the dedifferentiation of fibroblasts to a pluripotent state (52). Although little is known concerning the details of the interaction between Brg1 and STAT3 during LIF stimulation, recruitment of Brg1 occurs prior to or following STAT3 binding to its target genes, depending on the particular IL-6 target genes. For specific IL-6 target genes, Brg1 is constitutively bound and its presence is necessary for the recruitment of STAT3 (47). For other IL-6 target genes, STAT3 binds first to its DNA and then Brg1 is recruited depending on the presence of STAT3 (48).

### Ctr9 is a subunit of the Paf1 complex

The STAT3-Ctr9 interaction is highly significant for pluripotency of mESCs since Ctr9 indirectly induces multiple histone modifications, including trimethylation of Lys4 and Lys36, dimethylation of Lys79 on histone H3 and ubiquitination of histone H2B, all of which are important for gene activation (53). Consistent with this, the level of trimethylation of Lys4 on histone H3 on specific IL-6-inducible genes is dependent on the presence of Ctr9 (46). It appears that the interaction between STAT3 and Ctr9 is important for the recruitment of STAT3 to its target genes in this case. Although it is not known whether LIF also induces the interaction between STAT3 and Ctr9 in mESCs, confirmation is likely to provide the first molecular link between LIF and epigenetic modifications in ESCs. The Paf1 complex also binds to *Oct4* in mESCs (54,55), but it is unknown whether STAT3 is relevant to this binding.

### STAT6 target genes

STAT6 is a transcription factor and mainly responsible for their own transcriptional effects. It is primarily activated by IL-4 and IL-13 and its C-terminal Src homology 2 (SH2) domain is a specific phosphorylated receptor site. STAT6 is phosphorylated on Tyr641 and is subsequently activated. The STAT6 protein then dimerizes and translocates to the nucleus where it binds to STAT regulatory elements and regulates transcription in association with other transcription factors in its activated form (56). Previously, STAT6 has been reported to be constitutively activated in HL-derived cell lines (57).

Previously performed high-throughput sequencing of chromatin immunoprecipitated DNA has identified genes bound by STAT6. In addition, a previous study compared genes bound by STAT6 in wild-type and STAT6^−/−^ Th2 cells and these results were compared with epigenetic modifications across the genome (58). In the current study, H3K4me3 was colocalized with 60% of the binding sites for STAT6. Various permissive epigenetic marks were coincided with specific STAT6-bound regions and IL-4, Gata3, IL-24, phospholipase C δ 1 and homeodomain interacting protein kinase 2 were included in the corresponding genes (58). In an additional study, human Th2 cells were used and compared with the STAT6 binding to genes between cells where the expression of STAT6 was knocked down by RNA interference and cells with normal STAT6 expression (59). In the present study, a kinetic analysis was performed and the identity of STAT6-dependent genes during the Th2 polarization process was determined. It was found that 80% of IL-4 regulated genes were dependent on STAT6 at the 48-h time point. Specific genes regulated by STAT6 included Gata3, CRTH2, IL-24, lymphotoxin β, suppressor of cytokine signaling (SOCS) 1. A resource which may be used for future studies to define further roles of STAT6 in T and B cells is provided with high-throughput screening for STAT6-regulated genes. Emerging evidence shows that STAT6 functions in other immune cells, as well as other non-immune cells. STAT6 is likely to be significant in determining the nature of genes that are regulated by STAT6 in these tissues.

### STAT6-binding proteins and gene activation

STAT6 functions may be analyzed using mice with disrupted *Stat6* alleles. A previous mouse study demonstrated that STAT6 is critical for a number of responses in T cells, including the development of Th2 cells and IL-4-stimulated proliferative responses. The expression of Th2 cytokines, including IL-4, -5 and -13, was diminished in *Stat6*^−/−^ mice (60). The expression of Gata3, the master regulator of Th2 differentiation, may be regulated by STAT6 (61). STAT6 is also required for the development of IL-9-secreting T cells (62–64). The mechanisms by which STAT6 regulates T-cell proliferation include decreasing the expression of p27^Kip1^, a known cyclin-dependent kinase inhibitor, which may be at the transcriptional and post-translational levels (65,66). STAT6 is required for cytotoxic T2-cell differentiation, as the production of IL-4 and IL-5 is completely lost with STAT6 deficiency in CD8 cells (67). Overall, STAT6 is required for IL-4-stimulated T-cell functions.

In B cells, STAT6 promotes immunoglobulin class switching to IgE and IgG1, as well as promoting the expression of specific cell surface molecules responsible for antigen presentation by B cells (68). In a previous study, the levels of IgE were evidently reduced in STAT6-deficient mice when the mice were sensitized with antigen or infected with *N. brasiliensis* (61). No differences were identified in immunoglobulin class switching to IgG1 when *STAT6*^−/−^ and control mice were immunized with IgD, but the levels of IgG1 were reduced in STAT6-deficient mice, an infection model with *N. brasiliensis* or *S. mansoni* (69). The expression of several cell surface molecules responsible for antigen presentation by B cells is induced by IL-4, including MHC II, CD80 and CD86. Previous studies using *STAT6*^−/−^ B cells and mice expressing a constitutive form of STAT6 showed that the IL-4-mediated induction of these molecules is dependent on STAT6 (60,68). The expression of other cell surface molecules, such as CD23 and IL-4R-α, are also induced by STAT6 (68). CD23 is the low-affinity Fc receptor for IgE and is also a B-cell differentiation marker. The induction of IL-4R-α by STAT6 indicates that STAT6 promotes an autocrine-positive feedback loop for IL-4-dependent signaling. In B cells, STAT6 is required for IL-4-stimulated proliferation, similar to the previously described role of T cells (61). In addition, apoptosis is prevented in B cells by IL-4 in a STAT6-dependent manner (70).

STAT6 also functions in macrophages and dendritic cells, in addition to a requirement in T and B cells. STAT6 mediates IL-13-induced expression of genes, including MHC class II, and promotes IL-4-induced differentiation of alternatively activated macrophages (AAM) in macrophages (71,72). STAT6 activity in AAMs is associated with the suppression of T-cell proliferation (73). Currently, one previous study has shown that STAT6 facilitates the transcription mediated by the peroxisome proliferator-activated-γ receptor in macrophages and dendritic cells (74). STAT6 is capable of downregulating the production of IL-10 and promoting the production of IL-12 to promote a Th1 response in dendritic cells (75). Thus, STAT6 is vital in regulating the balance of inflammatory and allergic immune responses.

### STAT pathway in hematological malignancies

STAT proteins are activated by a wide range of cytokines and growth factors, typically via cytokines through the JAK family of tyrosine kinases (76).

Aprevious study revealed a close cross-talk correlation between STAT3 and phosphoinositide 3-kinase (PI3K) (77). STAT3 in PI3K-transformed murine cells is phosphorylated on Y705 and activated in a PI3K-dependent manner. Dominant-negative STAT3 interferes with PI3K-induced oncogenic transformation. In PI3K-transformed murine cells, phosphorylation of STAT3 is mediated by the TEC kinase, BMX. STAT3 is important in PI3K-driven oncogenic transformation and marks BMX as a promising therapeutic target that may enhance the efficacy of PI3K inhibitors. The PI3K-mammalian target of rapamycin and STAT3 signaling pathways represent two different regulatory networks. The functional link between these pathways is significant for the understanding of the PI3K- and STAT3-driven oncogenic mechanisms and identifies the TEC kinase, BMX, as a new cancer target.

STAT3 mutations unify the pathogenesis of chronic lymphoproliferative disorders of natural killer (NK) cells and T-cell large granular lymphocyte leukemia. STAT3 gene mutations are present in T and NK cell diseases. Mutations have been found in exons 21 and 20, encoding the SH2 domain. Constitutive STAT3, Tyr705 and Ser727 phosphorylation caused by the autocrine secretion of IL-6 exist in acute myeloid leukemia cells, murine plasmacytomas and hybridomas. In addition, STAT3-mediated constitutive expression of SOCS-3 is present in cutaneous T-cell lymphoma (78).

STAT3 is crucial in promoting the progression of hematological malignancies, including chronic lymphocytic leukemia (CLL). In CLL, STAT3 is constitutively phosphorylated on serine 727, regardless of blood count, disease stage or treatment status and not on the tyrosine 705 residue; however, the biological significance of serine-phosphorylated STAT3 (pSTAT3) is not known. A previous study demonstrated that constitutive serine pSTAT3 translocates to the nucleus by the karyopherin-β nucleocytoplasmic system and binds to DNA. Dephosphorylation of inducible tyrosine pSTAT3 did not affect STAT3-DNA binding. Furthermore, infection of CLL cells with lentiviral STAT3-small hairpin RNA (shRNA) reduced the expression of several STAT3-regulated survival and proliferation genes and induced apoptosis. Overall, the results suggested that constitutive phosphorylation of STAT3 on the serine 727 residue is a hallmark of CLL and that STAT3 may be considered a therapeutic target in this disease (79).

Targeting sphingosine-1-phosphate (S1P)/sphingosine-1-phosphate receptor 1 (S1PR1) using a clinically relevant and available drug or other approaches is potentially an effective, new therapeutic modality for treating the activated B cell-like subtype of diffuse large B-cell lymphoma, a subset of lymphoma that is less responsive to current available therapies. Studies have shown that using S1PR1 shRNA, a G protein-coupled receptor for S1P or FTY720, an antagonist of S1P that is used clinically for other indications, inhibits S1PR1 expression and downregulates STAT3 activity, causing growth inhibition of the B-cell lymphoma tumor cells *in vitro* and *in vivo* (80).

Recurrent mutations of the STAT6 DNA binding domain strongly support the involvement of STAT6 in the pathogenesis of this aggressive B-cell lymphoma (81). The STAT6 signaling pathway, activated by the cytokines IL-4 and IL-13, induces expression of the Epstein-Barr virus (EBV)-encoded protein LMP-1 in absence of the EBV nuclear antigen 2; implicating the type II EBV latent gene expression in HL (82).

## 4. Role of STAT3 and STAT6 in IL-9 production and its immune function

STAT3 is a central cytoplasmic transcription factor that is activated by the phosphorylation of a conserved tyrosine residue in response to oncogenic proteins and extracellular signals, such as cytokines and growth factors (83). STAT3 regulates a number of genes that are critical to tumor cell survival and proliferation, angiogenesis, invasion, metastasis and immune evasion (84,85). Previous extensive studies have demonstrated that inappropriate activation of STAT3 occurs at a high frequency in a wide variety of human cancers, including leukemia and lymphoma (86–89). Co-expression of the IL-9Rα chain promotes JAK1 mutant phosphorylation and STATs activation, including STAT1, STAT3 and STAT5.

**Figure 2:**
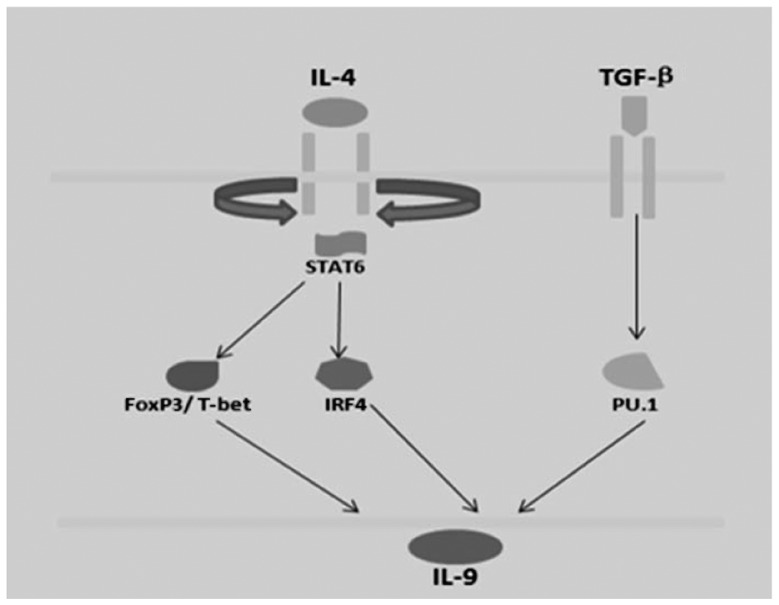
Association between IL-9 and STAT6. IL-9, interleukin-9; STAT6, signal transducer and activator of transcription 6; TGF, transforming growth factor; IRF4, interferon regulatory factor 4; FoxP3, forkhead box P3.

In addition, Th2 and Th9 cells are described as IL-9-secreting cells, and culture with TGF-β1 may increase IL-9 production. Th9 cells are derived in culture with a combination of TGF-β1 and IL-4. In addition, Th9 cells are associated with Th2 cells in that they require STAT6 and Gata3 for development, but exhibit reduced expression of Th2 cytokines. STAT6 is required for the expression of *Gata3* in Th9 cells (90). Ectopic expression of Gata3 reduced IL-9 production in wild-type cells but did not induce IL-9 production when transduced into *STAT6*^−/−^ Th9 cultures, suggesting that it is not directly regulating the *Il9* gene. It is possible that Gata3 is an intermediate in the STAT6-dependent reduction. IL-9 is not expressed by Th2 and Th9 cells in the absence of STAT6 expression (Fig. 2).

## 5. Conclusions and future directions

IL-9 is a multifunctional cytokine secreted by Th2 lymphocytes. The IL-9R and common γ chains associated with JAK1 and JAK3 trigger STAT-1, -3 and -5. In addition, dysregulated IL-9 response leads to autonomous cell growth and malignant transformation of lymphoid cells associated with constitutive activation of the JAK/STAT pathway *in vitro*. The most recent example of a Th subset that requires multiple balanced signals to develop is Th9 cells that secrete IL-9. Th9 cells develop following exposure to TGFβ and IL-4, whereas TGFβ alone promotes the differentiation of Treg cells and IL-4 stimulates Th2 development (91). The integration of the two signals result in a Th subset that exhibits lower Foxp3 expression than Treg cultures and lower Th2 cytokine production than Th2 cells, but increased production of IL-9. However, it remains unclear how the integration of each signal results in the unique Th9 phenotype. In any case, the role of IL-9 and STATs has important implications for pathophysiology and treatment in hematological malignancies.
